# Actionable and incidental neuroradiological findings in twins with neurodevelopmental disorders

**DOI:** 10.1038/s41598-020-79959-8

**Published:** 2020-12-29

**Authors:** Lynnea Myers, Mai-Lan Ho, Elodie Cauvet, Karl Lundin, Torkel Carlsson, Ralf Kuja-Halkola, Kristiina Tammimies, Sven Bölte

**Affiliations:** 1 Center of Neurodevelopmental Disorders (KIND), Division of Neuropsychiatry, Centre for Psychiatry Research, Department of Women’s and Children’s Health, Karolinska Institutet and Stockholm Health Care Services, Region Stockholm, Gävlegatan 22B, 113 30 Stockholm, Sweden; 2 Department of Nursing, Gustavus Adolphus College, 800 College Avenue West, St. Peter, MN USA; 3 Department of Radiology, Nationwide Children’s Hospital, 700 Children’s Drive-ED4, Columbus, OH USA; 4 PRIMA Child and Adult Psychiatry, Götgatan 71, 116 21 Stockholm, Sweden; 5 Department of Medical Epidemiology and Biostatistics, Karolinska Institutet, Nobels Väg 12A, Stockholm, Sweden; 6 Astrid Lindgren Children’s’ Hospital, Karolinska University Hospital, Region Stockholm, Bioclinicum J9:30, Akademiska Straket 1, Solna, Sweden; 7 Curtin Autism Research Group, School of Occupational Therapy, Social Work and Speech Pathology, Curtin University, Kent Street, Bentley, Perth, WA 6845 Australia; 8 Child and Adolescent Psychiatry, Stockholm Health Care Services, Region Stockholm, Gävlegatan 22B, 113 30 Stockholm, Sweden

**Keywords:** Neurodevelopmental disorders, Neuroscience

## Abstract

While previous research has investigated neuroradiological findings in autism spectrum disorder (ASD) and attention-deficit hyperactivity disorder (ADHD), the entire range of neurodevelopmental disorders (NDDs) has not yet been well-studied using magnetic resonance imaging (MRI). Considering the overlap among NDDs and simultaneous development of the brain and face, guided by molecular signaling, we examined the relationship of actionable and incidental (non-actionable) MRI findings and NDD diagnoses together with facial morphological variants and genetic copy number variants (CNVs). A cross-sectional study was conducted with a twin cohort 8–36 years of age (57% monozygotic, 40% dizygotic), including 372 subjects (46% with NDDs; 47% female) imaged by MRI, 280 with data for facial morphological variants, and 183 for CNVs. Fifty-one percent of participants had MRI findings. Males had a statistically significantly higher percentage of MRI findings (57.7%) compared with females (43.8%, p = 0.03). Twin zygosity was not statistically significantly correlated with incidence or severity of specific MRI findings. No statistically significant association was found between MRI findings and any NDD diagnosis or facial morphological variants; however, MRI findings were statistically significantly associated with the number of CNVs (OR 1.20, 95% CI 1.00–1.44, p = 0.05, adjusted OR for sex 1.24, 95% CI 1.03–1.50, p = 0.02). When combining the presence of MRI findings, facial morphological variants, and CNVs, statistically significant relationships were found with ASD and ADHD diagnoses (p = 0.0006 and p = 0.002, respectively). The results of this study demonstrate that the ability to identify NDDs from combined radiology, morphology, and CNV assessments may be possible. Additionally, twins do not appear to be at increased risk for neuroradiological variants.

## Introduction

No clinical recommendations exist for using brain magnetic resonance imaging (MRI) in the evaluation of neurodevelopmental disorders (NDDs), such as autism spectrum disorder (ASD) and attention-deficit hyperactivity disorder (ADHD)^1–3^. However, neuroimaging may be considered in individuals with NDDs based on the presence of co-morbidity or neurological findings on exam^4–6^. Preclinically, MRI research indicates some findings typical of ASD or ADHD on a group level^6–8^; however, results are not sufficiently specific or sensitive to be reliable biomarkers for diagnostic assessment. Additionally, MRIs in individuals with NDDs have shown higher frequencies of neuroradiological findings. For example, Erbetta et al.^9^ found minor brain alterations in 44% of individuals with ASD versus 22% in typically developing (TD), and Boddaert et al.^10^ reported MRI findings in 48% of participants with ASD. Conversely, some studies have shown similar prevalence rates of MRI findings in children with NDDs compared with children with TD. For example, Gupta et al.^11^ found comparable rates in children with ASD (25.5%) compared with children with TD (28.6%) and Vasa et al.^12^ found no statistically significant differences among children with ASD (11%) and ADHD (12.1%), compared with TD children (11.1%). In a study by Monterrey et al.^13^, 68% of twins with ASD, 71% of their unaffected co-twins, and 58% of TD twin controls had one or more incidental finding (uncommon, but non-actionable) on MRI. The authors suggested twins might be at increased risk for incidental neurological findings, especially males with ASD, and that these may originate in utero. The study had limitations, including an exclusive focus on ASD, despite known overlap with other NDDs^14,15^. In addition, the sample was largely skewed towards males, and the study did not examine incidental neuroradiological findings within twin pairs, limiting the possibility to determine their shared nature.

It’s thought that the “face predicts the brain”^16^, highlighting their simultaneous development in utero and the likelihood of neighboring body systems within the same embryologic field to show morbidity, driven by genetically controlled molecular signals^17^. Morphological variants in one’s physical appearance occur in the general population^18–20^ and in individuals with genetic syndromes^21,22^ and NDDs^23–27^. In our center, we found an increase of whole body morphological variants in NDDs, particularly ASD^28^. However, facial morphological variants alone were not associated NDD diagnoses^29^. Still, because of the potential relationship between an increased presence of morphological variants in individuals with variations in brain structure and/or genetic code, morphological variants in individuals with NDDs may still be important for further investigation.

We have previously studied rare and postzygotic de novo copy number variants (CNVs) in twins enriched for NDDs^30^. Rare CNVs were increased in areas where genes are located that are implicated in disorders of the nervous system in monozygotic twin pairs where at least one twin had ASD. Other studies support a relationship between CNVs and morphological variants. Engels et al.^31^ found atypical facial features in all participants with CNVs and intellectual disability (ID). Girirajan et al.^32^ found that individuals with ID had multiple congenital anomalies and higher frequencies of CNVs. Recently, Tammimies et al.^33^ found that individuals with ASD, morphological variants, and MRI findings were more likely to have higher diagnostic yields through genetic testing. Still, only a handful of studies have looked at the relationship between CNVs and neuroradiological findings in NDDs, with most of these investigating ASD-associated duplications and deletions at 16p11.2^34–36^.

This study expands on the study by Monterrey et al.^13^ to include a larger sample of twins with diagnoses of ASD, ADHD, and NDDs as an overarching diagnostic entity. The primary aim for our study was to examine actionable and incidental MRI findings by sex, zygosity, and NDD diagnosis. The secondary aim was to explore MRI findings in relation to facial morphological features and genetic information (i.e., CNVs). As biomarkers continue to be explored to identify any NDD, including neuroimaging genetics, we used regression models to we test if multilevel assessments together would yield biological information predictive of NDD outcomes.

## Subjects and methods

### Subjects

Participants were aged 8–36 years and recruited through the Roots of Autism and ADHD Twin Study in Sweden (RATSS)^37^. Monozygotic (MZ) and dizygotic (DZ) twins from Sweden with either TD or NDDs are recruited in RATSS to explore the genetic and environmental etiology of NDDs^37^. RATSS is approved by the Regional Swedish Ethical Review Board and written informed consent is collected from participants if over the age of 18 or parents/legally authorized guardians for participants who are minors or participants who are unable to provide consent (i.e., participants with neurodevelopmental disorders that prevent their ability to provide consent). Participants undergo an extensive data collection procedure, including biosampling, neuroimaging with MRI, medical photography, and psychodiagnostic assessments. All procedures were carried out in accordance with relevant guidelines and regulations. Only same sex pairs were used in this study due to potential anatomical differences between male and female brains. The final sample for this study comprised structural MRI findings in 372 participants, automated facial morphological assessments on 280 participants^29^, and genetic CNV data on 183 participants published earlier^30^. Table 1 describes participants with MRI scans (N = 372).

**Table 1:** Demographic information on sample with MRI performed (n = 372).

	TD	ASD	ADHD	NDD
Number of individuals	201	85	101	171
Zygosity (MZ:DZ)	131:64 (6 pending)	46:38 (1 pending)	42:56 (3 pending)	83:84
Sex ratio (Female:Male)	111:90	33:52	39:62	65:106
Age, mean (SD, range)	18.6 (6.2; 8–32)	15.1 (5.1; 8–31)	17.9 (6.1; 8–36)	14.9 (5.2; 8–36)
Facial morphological variants, median (25th and 75th IQR range; overall range)	2 (0, 3; 0–13)	1 (0.25, 3; 0–8)	1 (0, 3; 0–8)	1 (0, 3; 0–8)
Number of total CNVs, median (25th and 75th IQR range; overall range)	3 (2, 5; 1–10)	3 (2, 4; 1–7)	3 (2, 4; 1–6)	3 (2, 4; 1–7)
Number of rare CNVs, median (25th and 75th IQR range; overall range)	1 (1, 2; 0–7)	1 (1, 3; 0–5)	1 (0, 2; 0–5)	1 (0, 2; 0–5)
IQ, mean (SD, range)	102.3 (13.3; 69–131)	90.6 (20.8; 40–142)	93.5 (17.5; 40–128)	92.9 (18.0; 40–142)
**Other diagnoses**				
ASD: n (%)	–	–	41 (40.6)	85 (49.7)
ADHD: n (%)	–	41 (48.2)	–	101 (59.1)
ID: n (%)	–	14 (16.5)	10 (9.9)	19 (11.1)
Other NDD: n (%)	–	21 (24.7)	32 (31.7)	61 (35.7)
Psychiatric Diagnosis: n (%)	36 (17.9)	30 (35.3)	38 (37.6)	61 (35.7)

Unadjusted for NDD comorbidities.

*MRI *magnetic resonance imaging, *MZ *monozygotic, *DZ *dizygotic, *SD *standard deviation, *CNV *copy number variant, *IQR *interquartile range, *IQ *intelligence quotient, *TD *typically developing, *ASD *autism spectrum disorder, *ADHD *attention–deficit hyperactivity disorder, *ID *Intellectual Disability, *NDD *neurodevelopmental disorder. Individuals can have a diagnosis of both ASD and ADHD and therefore, would be represented in both columns in the table, as well as the column titled “NDD”. Examples of other NDDs include communication disorder, specific learning disorder, motor disorder, or other neurodevelopmental disorders. Examples of psychiatric diagnoses include depression, anxiety, eating disorders, and obsessive–compulsive disorder. Using the GEE model, statistically significant differences (p < 0.05) exist in age and IQ between those with TD and those with a diagnosis of ASD, ADHD, or any NDD. No statistically significant differences exist in facial morphological variants scores or number of total or rare CNVs between those with TD and those with a diagnosis of ASD, ADHD, or any NDD. Due to the comorbidity of NDD diagnoses and some participants having more than one NDD diagnosis, comparisons between groups of NDDs were not performed.

### Diagnostic and behavioral assessments

Participants received detailed psychodiagnostic assessments for ASD, ADHD and intelligence^37^, which resulted in diagnoses of NDDs, psychiatric disorders, or TD through a consensus process involving experienced clinicians. Twin pairs are classified as concordant or discordant for ASD, ADHD, and TD, and NDDs overall (based on presence of any NDD diagnoses, including ASD and/or ADHD).

### Magnetic resonance imaging (MRI)

MRI scans were performed using a 3 T MR750 GE scanner at the Karolinska Institutet Magnetic Resonance Center. Both T1 (Inversion Recovery Fast Spoiled Gradient Echo—IR-FSPGR, 3D-volume, 172 sagittal slices, 256 × 256, FOV 24, voxel size 1 mm^3^, flip angle 12, TR/TE 8200/3.2, using a 32-channel coil array) and T2 (T2-weighted Fluid Attenuated Inversion Recovery or FLAIR volume) images were obtained, along with resting-state functional MRI and diffusion tensor imaging (DTI). Scanning sessions lasted 50 min.

The MRI examinations were initially reviewed one of several clinical radiologists at the Karolinska Hospital and later, by one experienced, independent pediatric neuroradiologist (M.L.H.). The neuroradiologist (blinded to participants’ diagnoses and previous MRI readings) reviewed the anatomic (T1, T2, FLAIR, and DTI) imaging sequences for all participants. Ordinal scoring was used to assess the MRI findings (Supplemental File 1).

Subsequently, readings between the clinical radiologists and the independent neuroradiologist were compared, with discrepancies rated as 0 (both readers scored the case as normal, or either reader mentioned incidental findings outside the brain), 1 (both readers made the same finding in the brain, but used different terms to describe the finding, which did not affect management), and 2 (original reader missed finding or misclassified a finding in a manner that affected clinical management). Those with ratings of “0” and “1” were considered congruent, while only eight scans from participants were found to have meaningful differences (a rating of “2”), representing an agreement of nearly 98%. For the eight scans, the readings by the neuroradiologist were considered the gold standard and used in the analyses.

### Automated facial morphological assessments

Two-dimensional medical facial photos were taken of participants in RATSS at the Karolinska Hospital Medical Photography Lab. The facial photos were securely transferred to the Face2Gene web platform (FDNA; Boston, USA) to be analyzed for the presence of any facial morphological variants, with the results reported back to the research team using Human Phenotype Ontology^38^. A total of 56 unique facial morphological variants were identified. In a previous study by our research group^29^, Face2Gene was found to have high interrater agreement with in-person, clinical ratings of facial morphological variants performed by experienced clinical geneticists (78.3–100%).

### CNV calling

The CNV data and the procedure for CNV calling has been published previously in Stamouli et al.^30^. In short, saliva-derived DNA was used for genotyping with the Infinium PsychArray-24 v1.1 (Illumina Inc., San Diego, California, USA), followed with CNV calling using three different algorithms. CNVs called by at least two algorithms were annotated and their frequency within the control population was estimated using available cohorts. The presence and size of CNVs were determined for 200 participants overall in RATSS, of which 183 are included in this study.

### Statistical analyses

SPSS versions 26 and R version 3.3.2. were used. Information on the presence of MRI findings, total number of facial morphological variants, total number of CNVs (including rare CNVs), and diagnostic and demographic data available for each participant were used for the analyses. To account for our twin design, we computed linear (for continuous outcomes) and logistic (for dichotomous outcomes) regression models, fitted by generalized estimating equations (GEE)^39^ to determine within and between pair associations.. Results are described either using beta (ß) estimates for linear regressions or Odds Ratios (OR) for logistic regressions, both with 95% Confidence Intervals (CI) (Table 3).

Participant IQ and age were not significantly related to the presence of any MRI finding in the between-pairs model. Participant was significantly related to the presence of any MRI finding, so that males (57.7%) were more likely than females (43.8%, p = 0.03) to have an MRI finding (Supplementary Table 1). Gestational age at birth was examined in relationship to the presence of any MRI findings, both by the number of weeks gestation and a binary variable for gestational age: full-term (39 weeks or greater) or early-term or preterm (less than 39 weeks) (as defined by guidelines from The American College of Obstetricians and Gynecologists and Society of Maternal–Fetal Medicine, 2017)^40^. Neither variable was significantly associated with MRI findings (p = 0.80 and p = 0.17, respectively).

For the primary aim, the between pairs model was used to assess differences in the presence of any MRI finding by participant diagnosis and sex between those with TD and those with various diagnoses of NDDs (Table 2). For the secondary aim, we examined the presence of any MRI finding a binary outcome, where the presence of one or more MRI findings was coded as a “1” and no findings as a “0” with participants’ (i) sex, (ii) zygosity, and (iii) NDD diagnoses. An additional analysis using the twin pair zygosity was conducted to examine the concordance of MRI findings within twin pairs to determine how many twin pairs had the same types and degrees of findings on MRI (including pairs where both had non-findings). The relationship between zygosity and twin pairs with matched MRI findings was examined with a Chi-Square Test of Independence. Then, we examined the relationship between MRI finding and the independent variables of (i) the number of facial morphological variants and (ii) the number of CNVs. Finally, we computed a simultaneous Wald test for each NDD diagnosis with (i) the presence of any MRI finding, (ii) the number of facial morphological variants, and (iii) the number of CNVs in order to test the null hypothesis that all included covariates were equal to 0, and reported the p-value of this test. Alpha for all statistical tests was set at 0.05.

**Table 2:** Frequency of neuroradiological (MRI) findings in total sample, by typical development, and by neurodevelopmental disorder diagnosis.

Neuroradiological Finding	Total (n = 372) n (%)	TD (n = 201) n (%)	ASD (n = 85) n (%)	ADHD (n = 101) n (%)	Any NDD (n = 171) n (%)
Any MRI finding	190 (51.1)	104 (51.7)	40 (47.1)	51 (50.5)	86 (50.3)
**Atrophy**					
Mild	100 (26.9)	50 (24.9)	21 (24.7)	28 (27.7)	50 (29.2)
Severe	1 (0.3)	1 (0.5)	0 (0)	0 (0)	0 (0)
**Ventriculomegaly**					
Mild	20 (5.4)	13 (6.5)	2 (2.4)	6 (5.9)	7 (4.1)
Moderate	4 (1.1)	0 (0)	2 (2.4)	2 (2.0)	4 (2.3)
Cavum	7 (1.9)	5 (2.5)	1 (1.2)	2 (2.0)	2 (1.2)
Perivascular spaces-mild enlargement	16 (4.3)	8 (4.0)	6 (7.1)	5 (5.0)	8 (4.7)
Cyst	3 (0.8)	2 (1.0)	0 (0)	1 (1.0)	1 (0.6)
**Cortex**					
Cortex-focal abnormality	3 (0.8)	2 (1.0)	0 (0)	0 (0)	1 (0.6)
Cortex-diffuse abnormality	1 (0.3)	1 (0.5)	0 (0)	0 (0)	0 (0)
**Corpus callosum**					
Corpus callosum-low lying splenium	15 (4.0)	6 (3.0)	5 (5.9)	7 (6.9)	9 (5.3)
Corpus callosum-mild abnormality	4 (1.1)	1 (0.5)	0 (0)	1 (1.0)	3 (1.8)
Corpus callosum-severe abnormality	3 (0.8)	1 (0.5)	2 (2.4)	2 (2.0)	2 (1.2)
**White matter**					
White matter-mild loss	1 (0.3)	0 (0)	0 (0)	0 (0)	1 (0.6)
White matter-significant loss	5 (1.3)	2 (1.0)	2 (2.4)	2 (2.0)	3 (1.8)
Abnormal hippocampi	3 (0.8)	0 (0)	2 (2.4)	2 (2.0)	3 (1.8)
Abnormal basal ganglia	4 (1.1)	1 (0.5)	2 (2.4)	2 (2.0)	3 (1.8)
Abnormal brainstem	3 (0.8)	1 (0.5)	2 (2.4)	2 (2.0)	2 (1.2)
**Cerebellum**					
Cerebellum-minimal hypoplasia	42 (11.3)	23 (11.4)	6 (7.1)	10 (9.9)	19 (11.1)
Cerebellum-mega cisterna magna	8 (2.2)	4 (2.0)	2 (2.4)	2 (2.0)	4 (2.3)
Cerebellum-mild hypoplasia	17 (4.6)	7 (3.5)	4 (4.7)	9 (8.9)	10 (5.8)
Cerebellum-significant atrophy	3 (0.8)	1 (0.5)	2 (2.4)	2 (2.0)	2 (1.2)
**Chiari**					
Chiari-minimal tonsillar ectopia	30 (8.1)	23 (11.4)	2 (2.4	4 (4.0)	7 (4.1)
Chiari-mild tonsillar ectopia	12 (3.2)	8 (4.0)	2 (2.4)	0 (0)	4 (2.3)
Chiari-Chiari I malformation	3 (0.8)	2 (1.0)	0 (0)	0 (0)	1 (0.6)
Mass present	4 (1.1)	2 (1.0)	2 (2.4)	1 (1.0)	2 (1.2)

Table presents the number and percentage of MRI findings by TD and diagnoses of NDDs. Using the GEE model to compare the presence of any MRI finding by TD compared with ASD, ADHD, or any NDD diagnosis, no statistically significant differences (p > 0.05) existed. Those with TD had the highest percentage of any MRI finding (51.7%). Tests of difference for specific MRI findings (e.g., mild or severe ventriculomegaly, cavum, etc.) were not appropriate to conduct due to the small subsample sizes within these specific types and degrees of MRI findings.

## Results

### MRI findings in the sample

Fifty-one percent of all participants had a MRI finding, with the TD sample highest at 51.7%. However, the rate of MRI findings did not statistically significantly differ between those with TD versus those with ASD, ADHD, or any NDD diagnosis (all p > 0.05). Common MRI findings in the total sample included mild atrophy (26.9%), minimal hypoplasia in the cerebellum (11.3%), and minimal tonsillar ectopia (8.1%). Males had a statistically significantly higher percentage of findings (57.7%) compared with females (43.8%, p = 0.03).

Thirty-four twin pairs (18.3% of total sample; 19 MZ pairs, 14 DZ pairs, 1 pair pending zygosity) had exact matches on the same type and degree of MRI findings. If we also included twin pairs with exact matches on non-findings (where both twins did not have any findings on their MRIs), 104 pairs (55.9% of the total sample; 61 MZ pairs, 41 DZ pairs, 2 pairs pending zygosity) had exact matches. Using χ^2^ analyses, we found no statistically significant association between being MZ or DZ (zygosity) with the presence of MRI findings either in twin pairs with exact matches (p = 0.78), or in twin pairs with exact matches on either the presence of findings or non-findings (p = 0.76).

### MRI findings and NDD diagnoses

Among the NDD diagnoses, ADHD had the highest rate of MRI findings at 50.5%, followed by any NDD diagnosis at 50.3%, and ASD at 47.1% (Table 2). There were no associations between MRI finding (binary outcome variable) and any NDD diagnoses in the within or between pairs models, nor when looking at MZ or DZ twins separately (Table 3).

**Table 3:** Between and within pair associations: neuroradiological findings on MRI, facial morphological variants, copy number variants, and neurodevelopmental disorder diagnoses.

Outcome ~ independent variable		Between pair estimate			Within pair estimate			MZ only within pair estimate			DZ only within pair estimate		
		Beta	95% CI (beta)	p	Beta	95% CI (beta)	p	Beta	95% CI (beta)	p	Beta	95% CI (beta)	p
IQ ~ finding on MRI	No adjustments	1.49	− 2.40 to 5.37	0.45	5.60	1.32 to 9.87	0.01	3.26	− 1.76 to 8.29	0.20	8.42	1.36 to 15.48	0.02
	Adjusted for sex	1.66	− 2.26 to 5.57	0.41									
		OR	95% CI (OR)	p	OR	95% CI (OR)	p	OR	95% CI (OR)	p	OR	95% CI (OR)	p
Finding on MRI ~ # facial morphological variants	No adjustments	0.91	0.80 to 1.04	0.15	1.16	0.73 to 1.86	0.53	1.75	0.88 to 3.47	0.11	0.76	0.37 to 1.59	0.47
	Adjusted for sex	0.89	0.78 to 1.02	0.09									
Finding on MRI ~ # total CNVs	No adjustments	1.20	1.00 to 1.44	0.05									
	Adjusted for sex	1.24	1.03 to 1.50	0.03									
Finding on MRI ~ # rare CNVs	No adjustments	1.28	1.02 to 1.60	0.04									
	Adjusted for sex	1.29	1.01 to 1.64	0.04									
Any NDD ~ finding on MRI	No adjustments	0.94	0.60 to 1.49	0.80	0.58	0.23 to 1.49	0.26	0.38	0.10 to 1.42	0.15	1.00	0.29 to 4.04	> 0.99
	Adjusted for sex	0.85	0.53 to 1.37	0.51									
ASD ~ finding on MRI	No adjustments	0.81	0.48 to 1.37	0.43	.56	0.19 to 1.66	0.29	0.33	0.07 to 1.66	0.18	1.00	0.20 to 5.01	> 0.99
	Adjusted for sex	0.76	0.45 to 1.29	0.09									
ADHD ~ finding on MRI	No adjustments	0.97	0.57 to 1.64	0.91	.56	0.19 to 1.66	0.29	0.33	0.034 to 3.24	0.34	0.67	0.19 to 2.38	0.53
	Adjusted for sex	0.90	0.52 to 1.56	0.72									

*OR *odds ratio, # Number, *IQ *intelligence quotient, *ASD *Autism Spectrum Disorder, *ADHD *attention–deficit/hyperactivity disorder, *NDD *neurodevelopmental disorder, *CNVs *copy number variants, *MZ *monozygotic, *DZ *dizygotic.

Table presents the associations between dimensional and categorical variables and finding(s) on MRI. Sex was adjusted for in each of the analyses due to the statistically significant difference in MRI findings by sex; however, in within pair analyses, all factors shared between pairs are adjusted for, thus the estimate is already adjusted for sex since only same-sexed pairs are analyzed. A statistically significant association exists between the total number of CNVs and rare CNVs and a finding on an MRI, so that increasing numbers of CNVs and rare CNVs were associated with the presence of MRI findings. This appears to be an error in the table footnote. For some analyses, within-pair estimates, including within-pair estimates split by MZ or DZ twin pairs, were not conducted due to the small sample sizes and lack of variability in outcomes for the twin participants. Using a simultaneous Wald test, both ASD and ADHD diagnoses were statistically significantly correlated with the presence of increasing numbers of facial morphological variants, numbers of CNVs, and presence of MRI findings (p = 0.0006 for ASD, p = 0.002 for ADHD). However, no relationship was found between these variables and the diagnosis of any NDD as an overarching diagnostic category (p = 0.21).

### Relationship of MRI findings with facial morphological variants and the number of CNVs

No significant associations were found between the presence of MRI findings and number of facial morphological variants, nor when we adjusted for sex (OR 0.91, 95% CI 0.80–1.04, p = 0.15, adjusted OR 0.89, 95% CI 0.78–1.02, p = 0.09). Significant associations were found between the presence of MRI findings with both the total number of CNVs (OR 1.20, 95% CI 1.00–1.44, p = 0.05; adjusted OR for sex 1.24, 95% CI 1.03–1.50, p = 0.02) and with the total number of rare CNVs (OR 1.28, 95% CI 1.02–1.60, p = 0.04, adjusted OR for sex 1.29, 95% CI 1.01–1.64, p = 0.04) (Table 3).

### Relationship of NDD diagnosis with neuroradiological, morphological, and genetic variants

The number of facial morphological variants, MRI findings, and the number of CNVs did predict ASD diagnoses (p = 0.0006) and ADHD diagnoses (p = 0.002). However, no relationship was found to NDD as an overarching diagnostic category (p = 0.21).

## Discussion

This study is the first to examine the predictability of NDDs using combined biological data from MRI, facial morphological variants, and CNVs. We found that these assessments together may be valuable for identification of ASD and ADHD and this finding has potential to translate to clinical practice related to the diagnosis for NDDs. Within the model, the presence of MRI findings in particular predicted increasing numbers of CNVs, including the number of rare CNVs. In addition, males showed more MRI findings than females; however, there was no difference in the rates of matched MRI findings in twins who were MZ versus DZ.

We were unable to replicate the high rates of neuroradiological findings that were reported in by Monterrey et al.^13^, especially for twins diagnosed with ASD. However, similar to the Monterrey et al.^13^ study, we did find individual twins with TD had the highest rates of MRI findings compared with those with a diagnosis of ASD. Our study did identify a statistically significant difference for MRI findings in males compared with females, in alignment with Monterrey et al.^13^. In contrast to Monterrey et al.^13^, our study used a more stringent classification to determine concordance of MRI findings so that findings (and/or non-findings) within the twin pair needed to be an exact match in type and severity. For some analyses, Monterrey et al.^13^ counted the presence of any incidental finding within twin pairs as a concordant finding. Because incidental findings can be completely different, and spatially and etiologically unrelated, this assumption resulted in a very high calculated percentage of twins with concordance of MRI findings in the Monterrey et al.^13^ study. When we looked at the percentage of twins who had exact matches of types and severities of findings (and/or lack of any incidental findings), we did not find such high rates.

Recommendations vary on the inclusion of MRIs for individuals undergoing diagnosis for NDDs^1–6^; however, our results indicate that the use of MRIs in individuals with facial morphological variants, and CNVs in particular, may be warranted in the diagnosis of ASD and ADHD. Further studies are needed, and it is possible that using more detailed measures such as quantitative metrics from MRI scans and full body morphological assessments, may yield a higher degree of precision and information. Additionally, only a handful of studies have explored the relationship between CNVs and MRIs previously in NDDs^34–36^; therefore, further studies exploring this relationship are also needed to confirm the potential role CNVs may play in abnormal brain development.

Despite our study’s strengths, some limitations need to be addressed. Selection bias may have resulted in TD participants in our study who lacked NDD diagnoses, but who may have had other psychiatric or neurological disorders potentially resulting in higher rates of MRI findings in our sample compared with some previous studies. Sample sizes for individuals within each individual NDD diagnosis were small, potentially resulting in lack of significant findings for some analyses. Overall, we found a small number of MRI findings, even with a large sample size, which may have occurred due to recruitment of higher-functioning participants who were able to participate in the various study procedures. The age of participants was limited in this study to those greater than 8 years due to the recruitment methods used in the RATSS project overall, as well as the procedures performed in the RATSS that are either difficult or burdensome to perform in younger children. Although we lacked a singleton control group, the purpose of RATSS, from which the sample in this study is derived, is to generate novel hypotheses that can be tested subsequently in larger singleton samples, such as EU-AIMS LEAP^41,42^. Therefore, this is a plan for a subsequent study within a larger consortium context. Finally, due to the subjective nature of clinical MRI interpretations, individual errors cannot be ruled-out, though the use of multiple raters helps establish reliability.

In conclusion, both TD twins and those with NDDs had either similar or higher rates of MRI findings in comparison to previous studies on singletons^9–12^, the percentage of findings were higher in males, but individually, were not related to NDD diagnosis. We found higher numbers of CNVs associated with the presence of MRI findings. Both ASD and ADHD were predicted by the presence of MRI findings, the number of facial morphological variants, and the number of CNVs combined; thereby, providing some support for the use of MRI in the diagnosis of these disorders. Compared with the previous twin study by Monterrey et al.^13^, our rates of MRI findings in twins with ASD were lower. We also found no difference in the rates of the matched MRI findings in between MZ and DZ twins in our sample.

## Supplementary Information

Supplementary Information.

## References

[1] Filipek PA, Accardo PJ, Ashwal S (2000). Practice parameter: Screening and diagnosis of autism: Report of the Quality Standards Subcommittee of the American Academy of Neurology and the Child Neurology Society. Neurology.

[2] Volkmar F, Siegel M, Woodbury-Smith M (2014). Practice parameter for the assessment and treatment of children and adolescents with autism spectrum disorder. J. Am. Acad. Child Adolesc. Psychiatry.

[3] Johnson CP, Myers SM, American Academy of Pediatrics Council on Children With D (2007). Identification and evaluation of children with autism spectrum disorders. Pediatrics.

[4] Schaefer GB, Mendelsohn NJ, Professional Practice and Guidelines Committee (2013). Clinical genetics evaluation in identifying the etiology of autism spectrum disorders: 2013 guideline revisions. Genet. Med..

[5] Moeschler JB, Shevell M, Committee On Genetics (2014). Comprehensive evaluation of the child with intellectual disability or global developmental delays. Pediatrics.

[6] Cooper AS, Friedlaender E, Levy SE (2016). The implications of brain MRI in autism spectrum disorder. J. Child Neurol..

[7] Dougherty CC, Evans DW, Myers SM, Moore GJ, Michael AM (2016). A comparison of structural brain imaging findings in autism spectrum disorder and attention-deficit hyperactivity disorder. Neuropsychol. Rev..

[8] Rommelse N, Buitelaar JK, Hartman CA (2017). Structural brain imaging correlates of ASD and ADHD across the lifespan: A hypothesis-generating review on developmental ASD-ADHD subtypes. J. Neural Transm..

[9] Erbetta A, Bulgheroni S, Contarino V (2014). Neuroimaging findings in 41 low-functioning children with autism spectrum disorder: A single-center experience. J. Child Neurol..

[10] Boddaert N, Zilbovicius M, Philipe A (2009). MRI findings in 77 children with non-syndromic autistic disorder. PLoS ONE.

[11] Gupta S, Kanamalla U, Gupta V (2010). Are incidental findings on brain magnetic resonance images in children merely incidental?. J. Child Neurol..

[12] Vasa RA, Ranta M, Huisman TA, Pinto PS, Tillman RM, Mostofsky SH (2012). Normal rates of neuroradiological findings in children with high functioning autism. J. Autism Dev. Disord..

[13] Monterrey JC, Philips J, Cleveland S (2017). Incidental brain MRI findings in an autism twin study. Autism Res..

[14] Antshel KM, Zhang-James Y, Wagner KE, Ledesma A, Faraone SV (2016). An update on the comorbidity of ADHD and ASD: a focus on clinical management. Expert Rev Neurother.

[15] Lai MC, Kassee C, Besney R (2019). Prevalence of co-occurring mental health diagnoses in the autism population: A systematic review and meta-analysis. Lancet Psychiatry.

[16] Demyer W, Zeman W, Palmer CG (1964). The face predicts the brain: Diagnostic significance of median facial anomalies for holoprosencephaly (Arhinencephaly). Pediatrics.

[17] Marcucio R, Hallgrimsson B, Young NM. Facial morphogenesis: Physical and molecular interactions between the brain and the face. In *Craniofacial Development*. 1st edn (ed. Chai, Y.) (Academic Press, Cambrigde, 2015). 10.1016/bs.ctdb.2015.09.001 PMC6936956 26589930

[18] Marden PM, Smith DW, McDonald MJ (1964). Congenital anomalies in the newborn infant, including minor variations. A study of 4,412 babies by surface examination for anomalies and buccal smear for sex chromatin. J. Pediatr..

[19] Merks JH, Ozgen HM, Cluitmans TL (2006). Normal values for morphological abnormalities in school children. Am. J. Med. Genet. Part A.

[20] Ulovec Z, Sosić Z, Skrinjarić I, Catović A, Civljak M, Szirovicza L (2004). Prevalence and significance of minor anomalies in children with impaired development. Acta Paediatr..

[21] Starbuck J, Reeves RH, Richtsmeier J (2011). Morphological integration of soft-tissue facial morphology in Down Syndrome and siblings. Am. J. Phys. Anthropol..

[22] Ostermaier KK. Down syndrome: Clinical features and diagnosis. 2019. https://www.uptodate.com/contents/down-syndrome-clinical-features-and-diagnosis.

[23] Angkustsiri K, Krakowiak P, Moghaddam B (2011). Minor physical anomalies in children with autism spectrum disorders. Autism.

[24] Miles JH, Takahashi TN, Hong J (2008). Development and validation of a measure of dysmorphology: Useful for autism subgroup classification. Am. J. Med. Genet. A.

[25] Minahim D, Rohde LA (2015). Attention deficit hyperactivity disorder and intellectual giftedness: A study of symptom frequency and minor physical anomalies. Revista Brasileira de Psiquiatria.

[26] Ozgen Hellemann GS, de Jonge MV, Beemer FA, van Engeland H (2013). Predictive value of morphological features in patients with autism versus normal controls. J. Autism Dev. Disord..

[27] Ozgen Hellemann GS, Stellato RK (2011). Morphological features in children with autism spectrum disorders: A matched case-control study. J. Autism Dev. Disord..

[28] Myers L, Anderlid BM, Nordgren A (2017). Minor physical anomalies in neurodevelopmental disorders: A twin study. Child Adolesc. Psychiatry Ment. Health.

[29] Myers L, Anderlid BM, Nordgren A (2020). Clinical versus automated assessments of morphological variants in twins with and without neurodevelopmental disorders. Am. J. Med. Genet. A.

[30] Stamouli S, Anderlid BM, Willfors C (2018). Copy number variation analysis of 100 twin pairs enriched for neurodevelopmental disorders. Twin Res. Hum. Genet..

[31] Engels H, Brockschmidt A, Hoischen A (2007). DNA microarray analysis identifies candidate regions and genes in unexplained mental retardation. Neurology.

[32] Girirajan S, Brkanac Z, Coe BP (2011). Relative burden of large CNVs on a range of neurodevelopmental phenotypes. PLoS Genet..

[33] Tammimies K, Marshall CR, Walker S (2015). Molecular diagnostic yield of chromosomal microarray analysis and whole-exome sequencing in children with autism spectrum disorder. JAMA.

[34] Sonderby IE, van der Meer D, Djurovic S, *et al.* Engima-CNV: Unraveling the effects of brain structure of rare copy number variants involved in autism and other neurodevelopmental disorders. In *International Society for Autism Research 2019; Montreal, Canada* (2019).

[35] Owen JP, Bukshpun P, Pojman N (2018). Brain MR imaging findings and associated outcomes in carriers of the reciprocal copy number variation at 16p11.2. Radiology.

[36] Qureshi AY, Mueller S, Snyder AZ (2014). Opposing brain differences in 16p11.2 deletion and duplication carriers. J. Neurosci..

[37] Bölte S, Willfors C, Berggren S (2014). The roots of autism and ADHD twin study in sweden (RATSS). Twin Res. Hum. Genet..

[38] Köhler S, Carmody L, Vasilevsky N (2019). Expansion of the Human Phenotype Ontology (HPO) knowledge base and resources. Nucleic Acids Res..

[39] Zetterqvist J, Vansteelandt S, Pawitan Y, Sjolander A (2016). Doubly robust methods for handling confounding by cluster. Biostatistics.

[40] The American College of Obstetricians and Gynecologists, Society for Maternal-Fetal Medicine. Definition of Term Pregnancy. 579, November 2013 (reaffirmed 2017).

[41] Loth E, Charman T, Mason L (2017). The EU-AIMS Longitudinal European Autism Project (LEAP): Design and methodologies to identify and validate stratification biomarkers for autism spectrum disorders. Mol. Autism.

[42] Charman T, Loth E, Tillmann J (2017). The EU-AIMS Longitudinal European Autism Project (LEAP): Clinical characterisation. Mol. Autism.

