# Supplementary material for: Actionable and incidental neuroradiological findings in twins with neurodevelopmental disorders
Source: Sci Rep. 2020 Dec 29;10:22417. doi: 10.1038/s41598-020-79959-8 (PMC7772336; doi:10.1038/s41598-020-79959-8)
Supplement: Supplementary file 1 — Supplementary Information. [file 41598_2020_79959_MOESM1_ESM.docx]

Actionable and Incidental Neuroradiological Findings in Twins
with Neurodevelopmental Disorders

Lynnea Myers, PhD^1,2^, Mai-Lan Ho, MD^3^, Elodie Cauvet, PhD^1^, Karl Lundin^1^, MSc, Torkel Carlsson, MD^1,4^, Ralf Kuja-Halkola, PhD^5^, Kristiina Tammimies, PhD^1,6^ and Sven Bölte, PhD^1,7,8^

Supplemental File 1: Neuroradiological Findings Assessed in Study Participants using Ordinal Scoring

- Cerebral atrophy (0 none, 1 mild, 2 moderate, 3 severe);
- Ventriculomegaly^1^ (0 none, 1 mild, 2 moderate, 3 severe);
- Perivascular spaces (0 normal, 1 mild enlargement);
- Cystic lesions (0 none, 1 cavum anomaly, 2 arachnoid cyst);
- Cortex (0 normal, 1 focal abnormality, 2 diffuse abnormality);
- Corpus callosum (0 normal, 1 low lying splenium, 2 mild abnormality, 3 severe abnormality);
- White matter volume (0 normal, 1 mild loss, 2 significant loss);
- Hippocampi (0 normal, 1 abnormal); basal ganglia (0 normal, 1 abnormal);
- Brainstem (0 normal, 1 abnormal);
- Cerebellum (0 normal, 1 minimal hypoplasia, 2 mega cisterna magna, 3 mild hypoplasia, 4 significant atrophy);
- Cerebellar tonsils (0 none, 1 minimal tonsillar ectopia, 2 mild tonsillar ectopia, 3 Chiari I malformation);
- Benign masses (0 none, 1 present).

^1^ Further confirmed using published ventricular measurements (Sari et al., 2015), including Evans’ index (maximal frontal horn ventricular width divided by the transverse inner diameter of the skull).

Reference:

Sari, E., Sari, S., Akgun, V., Ozcan, E., Ince, S., Babacan, O., Saldir, M., Acikel, C., Basbozkurt, G., Yesilkaya, S., Kilic, C., Kara, K., Vurucu, S., Kocaoglu, M., & Yesilkaya, E. (2015). Measures of ventricles and Evans' index: from neonate to adolescent. *Pediatric Neurosurgery, 50*(1), 12-17. <https://doi.org/10.1159/000370033>

**Supplemental Table 1: Neuroradiological Findings by Sex**

| **Incidental Finding** | **Sex** | |
| --- | --- | --- |
|  | Male  (n=196)  n (%) | Female  (n=176)  n (%) |
| Any MRI Finding | 113 (57·7) | 77 (43·8) |
| Atrophy | | |
| Mild | 61 (31·1) | 39 (22·2) |
| Severe | 1 (0·5) | 0 (0) |
| Ventriculomegaly | | |
| Mild | 15 (7·7) | 5 (2·8) |
| Moderate | 0 (0) | 4 (2·3) |
| Cavum | 3 (1·5) | 4 (2·3) |
| Perivascular Spaces- Mild Enlargement | 6 (3·1) | 10 (5·7) |
| Cyst | 3 (1·5) | 0 (0) |
| Cortex | | |
| Cortex-Focal Abnormality | 2 (1·0) | 1 (0·6) |
| Cortex-Diffuse Abnormality | 1 (0·5) | 0 (0) |
| Corpus Callosum | | |
| Corpus Callosum- Low Lying Splenium | 12 (6·1) | 3 (1·7) |
| Corpus Callosum- Mild Abnormality | 3 (1·5) | 1 (0·6) |
| Corpus Callosum- Severe Abnormality | 1 (0·5) | 2 (1·1) |
| White Matter | | |
| White Matter- Mild Loss | 1 (0·5) | 0 (0) |
| White Matter- Significant Loss | 2 (1·0) | 3 (1·7) |
| Abnormal Hippocampi | 0 (0) | 3 (1·7) |
| Abnormal Basal Ganglia | 1 (0·5) | 3 (1·7) |
| Abnormal Brainstem | 1 (0·5) | 2 (1·1) |
| Cerebellum | | |
| Cerebellum- Minimal Hypoplasia | 24 (12·2) | 18 (10·2) |
| Cerebellum- Mega Cisterna Magna | 6 (3·1) | 2 (1·1) |
| Cerebellum- Mild Hypoplasia | 9 (4·6) | 8 (4·5) |
| Cerebellum- Significant Atrophy | 1 (0·5) | 2 (1·1) |
| Chiari | | |
| Chiari- Minimal Tonsillar Ectopia | 14 (7·1) | 16 (9·1) |
| Chiari- Mild Tonsillar Ectopia | 6 (3·1) | 6 (3·4) |
| Chiari- Chiari I Malformation | 0 (0) | 3 (1·7) |
| Mass Present | 4 (2·0) | 0 (0) |

Table presents the number and percentage of neuroradiological MRI findings by sex. Using the GEE model to compare the presence of any MRI finding by sex, significant differences (p=·03) existed, with males having the higher percentage of any MRI finding (57·7%) compared with females (43·8%). Tests of difference by sex for specific MRI findings (e.g., mild or severe ventriculomegaly, cavum, etc.) were not appropriate to conduct due to the small subsample sizes within these specific types and degrees of MRI findings.
